# TRENDS IN HEALTHCARE COSTS AND UTILIZATION ASSOCIATED WITH HEARING LOSS DIAGNOSIS OVER 10 YEARS

**DOI:** 10.1093/geroni/igz038.1832

**Published:** 2019-11-08

**Authors:** Nicholas Reed, Jennifer A Deal, Frank Lin, Charlotte Yeh

**Affiliations:** 1 Johns Hopkins University, Baltimore, Maryland, United States; 2 Johns Hopkins University Bloomberg School of Public Health, Baltimore, Maryland, United States; 3 AARP Services, Inc., Washington, District of Columbia, United States

## Abstract

Hearing loss affects 38 million Americans and is associated with cognitive and physical decline. Moreover, hearing loss limits communication between patients and providers. In this study, we aimed to determine whether hearing loss diagnosis impacts healthcare cost and utilization. In the OptumLabs Data Warehouse insurance claims database (January 1, 1999 to December 31, 2016) we identified cases of age-related hearing loss (i.e. excluding conductive, ototoxic-induced, surgical-related hearing losses) in adults over the age of 50 years. Hearing loss cases were propensity-matched (nearest neighbor) to persons without hearing loss on multiple socioeconomic (income, region), demographic (age, sex, race, education), and health status (comorbidity count, cancer, dementia, baseline resource utilization) indices. There were 154,414, 44,852, and 4,728 subjects, respectively, at the 2-, 5-, and 10-year follow-up time points. We used regression modeling to examine healthcare cost (total expenditures), inpatient hospitalization, length of inpatient stay, outpatient visits, 30-day readmissions, and emergency department visits. Among 4,728 matched adults, hearing loss was associated with $22,434 (95% CI $18,219-$26,648) or 46% higher total healthcare costs over a 10-year period compared to those without hearing loss. Persons with hearing loss experienced more inpatient stays (Incident Rate Ratio, 1.47; 95% CI 1.29-1.68) and were at-risk (44% higher risk) for greater 30-day hospital readmission (Relative Risk, 1.44; 95% CI 1.14-1.81) at 10-years post index date. Similar trends were observed at 2- and 5-year time points across all measures. Importantly, these pathways may be amendable to hearing treatment via amplification which warrants future research preventative strategies.

